# Evaluation Methods, Indicators, and Outcomes in Learning Health Systems: Protocol for a Jurisdictional Scan

**DOI:** 10.2196/57929

**Published:** 2024-12-06

**Authors:** Shelley Vanderhout, Marissa Bird, Antonia Giannarakos, Balpreet Panesar, Carly Whitmore

**Affiliations:** 1 Institute for Better Health Trillium Health Partners Mississauga, ON Canada; 2 Institute of Health Policy, Management, and Evaluation University of Toronto Toronto, ON Canada; 3 Library & Knowledge Services Trillium Health Partners Mississauga, ON Canada; 4 School of Nursing Faculty of Health Sciences McMaster University Hamilton, ON Canada

**Keywords:** learning health systems, evaluation, jurisdictional scan, counterfactuals, LHS, health system, real-time evidence, informatics, organizational culture, learning cycles, benchmark, patient care, gaps, health care, inequities, development, implementation, intervention, new approach

## Abstract

**Background:**

In learning health systems (LHSs), real-time evidence, informatics, patient-provider partnerships and experiences, and organizational culture are combined to conduct “learning cycles” that support improvements in care. Although the concept of LHSs is fairly well established in the literature, evaluation methods, mechanisms, and indicators are less consistently described. Furthermore, LHSs often use “usual care” or “status quo” as a benchmark for comparing new approaches to care, but disentangling usual care from multifarious care modalities found across settings is challenging. There is a need to identify which evaluation methods are used within LHSs, describe how LHS growth and maturity are conceptualized, and determine what tools and measures are being used to evaluate LHSs at the system level.

**Objective:**

This study aimed to (1) identify international examples of LHSs and describe their evaluation approaches, frameworks, indicators, and outcomes; and (2) describe common characteristics, emphases, assumptions, or challenges in establishing counterfactuals in LHSs.

**Methods:**

A jurisdictional scan, which is a method used to explore, understand, and assess how problems have been framed by others in a given field, will be conducted according to modified PRISMA (Preferred Reporting Items for Systematic Reviews and Meta-Analyses) guidelines. LHSs will be identified through a search of peer-reviewed and gray literature using Ovid MEDLINE, EBSCO CINAHL, Ovid Embase, Clarivate Web of Science, PubMed non-MEDLINE databases, and the web. We will describe evaluation approaches used both at the LHS learning cycle and system levels. To gain a comprehensive understanding of each LHS, including details specific to evaluation, self-identified LHSs will be included if they are described according to at least 4 of 11 prespecified criteria (core functionalities, analytics, use of evidence, co-design or implementation, evaluation, change management or governance structures, data sharing, knowledge sharing, training or capacity building, equity, and sustainability). Search results will be screened, extracted, and analyzed to inform a descriptive review pertaining to our main objectives. Evaluation methods and approaches, both within learning cycles and at the system level, as well as frameworks, indicators, and target outcomes, will be identified and summarized descriptively. Across evaluations, common challenges, assumptions, contextual factors, and mechanisms will be described.

**Results:**

As of October 2024, the database searches described above yielded 3503 citations after duplicate removal. Full-text screening of 117 articles is complete, and 49 articles are under analysis. Results are expected in early 2025.

**Conclusions:**

This research will characterize the current landscape of LHS evaluation approaches and provide a foundation for developing consistent and scalable metrics of LHS growth, maturity, and success. This work will also serve to identify opportunities for improving the alignment of current evaluation approaches and metrics with population health needs, community priorities, equity, and health system strategic aims.

**Trial Registration:**

Open Science Framework b5u7e; https://osf.io/b5u7e

**International Registered Report Identifier (IRRID):**

DERR1-10.2196/57929

## Introduction

Evidence-to-practice gaps in health care contribute to inefficient and ineffective care, ballooning costs, poor experiences for patients, frustration and burnout for health care providers, and widening health inequities [1]. In response to these challenges, there is a growing emphasis on the development, implementation, and evaluation of learning health systems (LHSs). LHSs address gaps between knowledge and practice by combining real-time evidence, informatics systems, patient-provider partnerships and experiences, and institutional strategies in learning cycles, which support continuous innovation and improvements in care [2]. Worldwide, various health care organizations have adopted LHS approaches, and while the objectives are often consistent, evaluation methods (both within learning cycles and at the system level) and markers of growth, maturity, and success tend to be more variable and incomprehensively described [3,4]. Evaluation approaches within learning cycles of LHSs, and of LHSs themselves, often depend on factors such as data availability, quality, and sharing restrictions (particularly for routinely collected data to inform baseline measures), context, time, experimental methodology, and knowledge translation [5,6]. This results in variable feasibility, applicability, and relevance across settings and presents a challenge for identifying what a “successful” LHS looks like in practice; determining the overall effectiveness of LHS on patient, provider, population, cost, and equity outcomes; and continuously improving how LHSs operate.

When LHSs conduct cycles of data collection, knowledge synthesis, and practice change [7], they often use “usual care” or “status quo” as the benchmark or counterfactual for comparing new interventions or approaches. However, in the evolving landscape of pragmatic and realist research, teasing apart what is “usual” or what would happen if an event or condition had been different has become a challenging endeavor as a result of the complexity of the system and subsequent interventions. This is particularly a challenge when attempting to disentangle components that contribute to success in new care solutions from the multifarious existing care modalities found across settings [5]. As a result, empirical evidence has occasionally revealed unexpected outcomes when comparing complex care models or approaches to usual care, wherein, for example, the introduction of novel elements, such as health care worker interventions featuring more frequent touchpoints and early health challenge detection, can paradoxically result in adverse outcomes, such as increased hospital admissions [8,9]. Assessing the impact of new care models in an LHS should be assessed holistically, including investigation into not just “what works” but also questions about “for whom, under which circumstances, and why?” [10].

Despite best efforts to share methodological innovations and lessons learned specific to LHSs, a comprehensive scan of existing LHS evaluation approaches is needed. This protocol describes a jurisdictional scan to scope and characterize the evaluation approaches and methodologies in international examples of self-identified LHSs with the following objectives:

1. Identify international examples of LHSs and describe their evaluation approaches, frameworks, indicators, and outcomes
2. Elucidate common characteristics, emphases, assumptions, or challenges described in establishing counterfactuals in LHS research, and describe how these counterfactuals impact the evaluation process

## Methods

### Overview

Jurisdictional scans are used to explore and understand how problems have been framed by others in a given field and to compare and assess the strengths and weaknesses of each approach [11]. In addition, they aim to produce policy-relevant results by including details of the problem in context, making them useful tools for understanding how a specific initiative has been framed, conducted, or disseminated in other jurisdictions [12]. This approach was selected over others such as a narrative review, which provides a detailed description of a topic but without an emphasis on policy relevance, or a realist review, which seeks to understand contexts and mechanisms associated with intervention outcomes due to its use for performing comparative analyses, identifying practical implementation insights, and highlighting context-specific information. This approach is fitting for LHSs because the concept of an LHS is often interpreted, envisioned, and constructed differently across contexts due to its relative novelty; variety of frameworks available; and context-driven priorities, resources, and leadership [13]. The results of this jurisdictional scan will summarize insights, key learnings, challenges, and implementation considerations with attention to both context-specific factors and generalizability across settings. This jurisdictional scan will use a literature review to identify potentially relevant LHSs.

### Theoretical Approach

**Figure 1:**
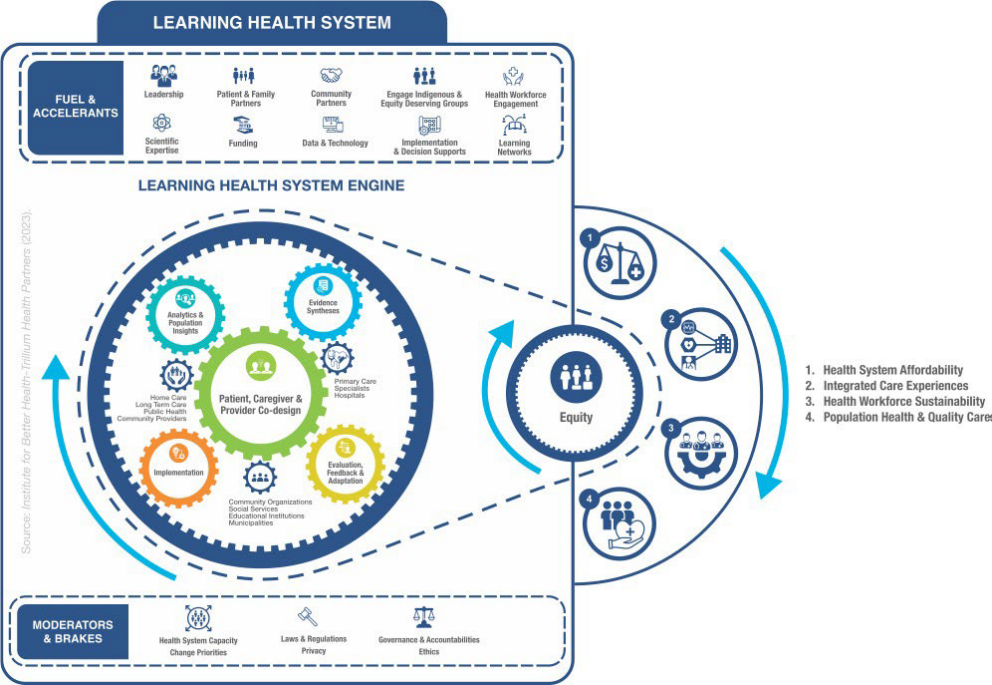
Learning Health System Action Framework (reproduced from Reid et al [13], which is published under Creative Commons Attribution 4.0 International License [14]).

The LHS Action Framework [13,14] (Figure 1) informs the authors’ approach to understanding LHSs. This framework was developed by consolidating existing LHS frameworks in the literature, and it describes how research and health care operations are linked and enacted in a comprehensive LHS approach to advance population health and health equity. It was produced to identify capabilities necessary to enact the learning elements required, including key questions and methods, to ensure a systematic approach to learning and achieve equity-centered quadruple aim metrics. The LHS Action Framework has five learning gears: (1) analytics and population insights; (2) evidence synthesis; (3) patient, caregiver, and provider co-design; (4) implementation; and (5) evaluation, and three health system gears representing different care settings, services, and institutions. In this review, the focus is on the evaluation gear while acknowledging the relevance to and impact of the other 4 gears on LHS evaluation.

The Medical Research Council (MRC) Framework for Developing and Evaluating Complex Interventions [10] will guide the assessment of how each LHS conducts evaluation. LHSs, either at the learning cycle or system level, are complex interventions as they often involve implementing and integrating multiple and diverse components and targeting a range of behaviors among different populations and settings, and require broad and varied expertise and skills [10]. The MRC framework not only guides consideration for whether an intervention achieves its intended outcome but adds relevant context such as value relative to required resources, peripheral intended or unintended impacts, theoretical underpinnings, interactions with the broader environment, and contributions to system change and decision making. This framework is particularly relevant to LHSs as it also focuses on the individuals involved in determining evaluation questions and outcomes and how those individuals are impacted by an intervention, consistent with a core pillar of LHS models that emphasize patient and health care provider perspectives [2,13].

In absence of specific reporting guidance for jurisdictional scans, the authors adapted the PRISMA-ScR (Preferred Reporting Items for Systematic reviews and Meta-Analyses extension for Scoping Reviews) checklist (Multimedia Appendix 1). An information specialist (AG) with expertise in LHSs and international terminology used to describe them supported the search development and implementation across Ovid MEDLINE, EBSCO CINAHL, Ovid Embase, Clarivate Web of Science, and PubMed non-MEDLINE databases, as well as gray literature and relevant websites (the search strategy is shown in Multimedia Appendix 1). The search strategy was built based on the search by Enticott et al [15] and defines LHSs according to the Institute of Medicine’s [16] definition, “a system in which progress in science, informatics, and care culture align to generate new knowledge as an ongoing, natural by-product of the care experience, and seamlessly refine and deliver best practices for continuous improvement in health and healthcare.” Authors will also leverage professional networks to identify relevant sources of literature in the form of websites, newsletters, or online or print reports.

#### Eligibility Criteria

LHSs that are self-described as such in peer-reviewed journal articles, reports, and web pages will be considered for inclusion. Authors must self-identify their LHS using at least one of the following descriptors, which were informed by existing literature [17] to ensure international and context-specific terminology for LHS were captured:

1. Learning health[care] system
2. Learning health[care] network
3. Learning collaborative
4. Learning laboratory
5. Community-clinician participatory data health care research
6. Data-driven improvement initiative
7. Practice-based data or research network
8. Circular data-driven health care
9. Rapid LHS

Only literature published after 2007, when LHSs were formally conceptualized [18], and those written in English will be considered. LHSs will only be included when described in sufficient detail according to at least 4 of 11 criteria that describe LHSs, which were informed by the LHS Action Framework and other literature [4,13,19] (core functionalities, analytics, use of evidence, co-design or implementation, evaluation, change management or governance structures, data sharing, knowledge sharing, training or capacity building, equity, and sustainability), to facilitate a fulsome description of the system within the resultant papers. Given that there is a great deal of ambiguity in how LHSs are defined and described, broad inclusion criteria were chosen based on a review of candidate papers, discussions with leaders in this topic, and team knowledge of varied terminology used to describe LHSs across regions and contexts. Therefore, an LHS described only as a research setting, for example, but without further information will not be included. Multiple publications about a single LHS may be combined to pool information about these characteristics if needed; reference lists of each included paper will be reviewed to identify complementary articles. Ideally, each identified LHS will include a description of its evaluation approach either at learning cycle or system levels, but articles that describe LHSs without information about evaluation will still be included to highlight gaps in the literature where they may exist.

A total of 3 independent screeners (SV, CW, and MB) will review all titles and abstracts identified in the literature search in Covidence in duplicate. Authors SV, CW, and MB will resolve any uncertainties and verify the final sample. A data extractor (BP) will independently code each LHS according to the 11 possible constructs listed above, which will then be reviewed by the study team (SV, CW, and MB) to verify accuracy and reach a consensus for inclusion. Team meetings will be held biweekly to review progress, resolve queries, and troubleshoot issues.

### Data Extraction

Each included LHS will be described according to the following core evaluation characteristics, where data are available. For a full description of extraction criteria definitions, refer to Table S1 in Multimedia Appendix 1.

- LHS objectives
- At the learning cycle level:
  Evaluation, feedback, and adaptation approaches, mechanisms, and methods
  Data sources, including counterfactuals
  How counterfactuals are established and defined
- At the system level:
  Evaluation methods
  Evaluation framework
  Metrics, indicators, or outcomes of growth, maturity, or success
  Strategies for routine data collection, quality, and accessibility or sharing
  Evaluation data sources
- Change management and governance structures
- Contextual factors (eg, system priorities, breadth, and resources; time; and clinical population)
- Data and infrastructure sharing processes
- Personnel involved in evaluation
- Knowledge sharing practices

Additional descriptors, where available, will include:

- LHS name
- Country
- Sector(s) involved (primary care; hospital; specialist care; long-term care; community, population, or public health; industry; and other)
- Patient population(s) served
- Patient population size
- Personnel involved (patients, health care providers, operational staff, leadership, and other)
- Reference to grounding framework or model or theoretical development

### Analysis

Data analysis for this review will follow a 2-stage process aimed at addressing our two research objectives, which are (1) to identify international LHS and describe their evaluation approaches, frameworks, indicators, and outcomes, and (2) to determine common facilitators and challenges described in establishing counterfactuals in LHS research. A combination of quantitative (ie, frequency counts) and qualitative (ie, thematic analysis of publication text) methods will be used to summarize findings relevant to each objective. From these findings, we will generate a summary of recommendations for evaluation approaches and future research, aimed at both individual LHS learning cycles and LHSs more broadly. It is anticipated that this analysis process may also yield gaps in the literature, especially on the establishment of counterfactuals in LHS research. Regardless of the comprehensiveness of our findings, we will discuss the implications of what was not reported and provide recommendations of where future work can be focused in order to strengthen the field of LHSs. The data extraction form is shown in Multimedia Appendix 1. All data will be extracted into a spreadsheet through Airtable (Formagrid, Inc) [20] and descriptors will be summarized as appropriate.

### Patient Partnership and Knowledge Translation Plan

Using the Knowledge-to-Action framework by Graham et al [21], authors will develop an active knowledge translation plan by (1) identifying key messages arising from this scan, (2) determining the principal target audience for each of these messages, (3) seeking and involving the most credible messenger for these messages, and (4) launching a knowledge translation strategy that is grounded in the best available evidence. Drawing upon a diverse range of approaches to disseminate the results of this scan, including a virtual symposium that will bring together the key target audience of this research, publishing our results, and presenting findings at local and international scientific conferences, these strategies will ensure that these findings reflect the needs of the end users of this information and facilitate appropriate sharing of outputs. The study authors lead an international LHS collaboration hub and will co-design the next steps, guided by the findings of this work, alongside multidisciplinary health system leaders, patient and community partners, clinicians, and policy makers.

### Anticipated Challenges

There may be potential challenges related to this jurisdictional scan. First, the yield of the literature search may be more extensive than anticipated. To overcome this, authors will work closely with the information specialist to ensure that the scope of the scan is manageable but comprehensive. Second, it is anticipated that for many LHSs, it will be challenging to discern or categorize evaluation characteristics solely from what is described and published. Corresponding authors of published literature will be contacted through email to obtain additional details when necessary.

## Results

As of October 2024, the database searches described above yielded 3503 citations after duplicate removal. Full-text screening of 117 articles is complete, and 49 articles are under analysis. Results are expected in early 2025.

## Discussion

### Expected Findings

This jurisdictional scan will serve to map and characterize existing self-identified international LHSs and provide an improved understanding of how these LHSs conduct evaluations both within learning cycles and at the system level. Many LHSs worldwide have not yet reached full maturity or may not have established fulsome evaluation approaches [2,22], which adds complexity to this work but will help identify opportunities for improvement, collaboration, and resource development. Although available literature does report on common LHS objectives and how they are operationalized [4,23], approaches to comprehensive LHS evaluations at the system and learning cycle level have not yet been identified, summarized, and presented. Furthermore, once described, there is a need to understand what constitutes success of adopting an LHS approach, how it is attained and maintained, and what would happen if the LHS had not been implemented. This study will address this research gap.

This jurisdictional scan may have some limitations. While we will use a comprehensive search strategy developed by a research librarian, the reliance on self-report to identify LHSs means that this review may not identify or capture all LHSs and their evaluation approaches, especially as some may be unreported or documented internally within health systems. We also anticipate considerable variation in the approach and scale of LHS evaluations, which may make data synthesis challenging; however, this study is intended to be descriptive and will provide an evidence base that may guide more consistent evaluation approaches in the future.

### Conclusions

By describing how LHSs currently conceptualize success and measure impacts on patient, provider, population, cost, and equity outcomes, knowledge generated from this research will contribute to the development of harmonized criteria health system leaders, researchers, and community partners can use to benchmark progress of a maturing LHS and set context-relevant targets for evaluation, growth, and improvement. This work will also serve to identify opportunities for improving the alignment of current evaluation approaches and metrics with population health needs, community priorities, and health system strategic aims. We will leverage a Canadian network of health system leaders, clinicians, researchers, trainees, and patient and community partners to communicate and build upon the findings of this work, which will increase its potential to be used as foundation for building evaluation tools, frameworks, and resources.

## Appendix

Multimedia Appendix 1 PRISMA-ScR (Preferred Reporting Items for Systematic Reviews and Meta-Analyses Extension for Scoping Reviews) checklist, search strategy, data extraction form, and extraction criteria and definitions.

## Abbreviations

LHS: learning health system
MRC: Medical Research Council
PRISMA: Preferred Reporting Items for Systematic Reviews and Meta-Analyses
PRISMA-ScR: Preferred Reporting Items for Systematic Reviews and Meta-Analyses Extension for Scoping Reviews

## Data Availability

No primary data have been generated or analyzed at this stage. All data sources will be properly cited and referenced in the final publication. The datasets analyzed during this scan will be available from the corresponding authors of the original studies. Any supplementary materials or datasets generated during the review process will be made available on study completion with reasonable request.
