# Supplementary material for: Evaluation Methods, Indicators, and Outcomes in Learning Health Systems: Protocol for a Jurisdictional Scan
Source: JMIR Res Protoc. 2024 Dec 6;13:e57929. doi: 10.2196/57929 (PMC11694705; doi:10.2196/57929)
Supplement: Multimedia Appendix 1 [file resprot_v13i1e57929_app1.docx]

**PRISMA-ScR (Preferred Reporting Items for Systematic reviews and Meta-Analyses extension for Scoping Reviews) Checklist**

| **SECTION** | **ITEM** | **PRISMA-ScR CHECKLIST ITEM** | **REPORTED ON PAGE #** |
| --- | --- | --- | --- |
| **TITLE** | | | |
| Title | 1 | Identify the report as a scoping review. | 1 |
| **ABSTRACT** | | | |
| Structured summary | 2 | Provide a structured summary that includes (as applicable): background, objectives, eligibility criteria, sources of evidence, charting methods, results, and conclusions that relate to the review questions and objectives. | 2 |
| **INTRODUCTION** | | | |
| Rationale | 3 | Describe the rationale for the review in the context of what is already known. Explain why the review questions/objectives lend themselves to a scoping review approach. | 3 |
| Objectives | 4 | Provide an explicit statement of the questions and objectives being addressed with reference to their key elements (e.g., population or participants, concepts, and context) or other relevant key elements used to conceptualize the review questions and/or objectives. | 4 |
| **METHODS** | | | |
| Protocol and registration | 5 | Indicate whether a review protocol exists; state if and where it can be accessed (e.g., a Web address); and if available, provide registration information, including the registration number. | 5 |
| Eligibility criteria | 6 | Specify characteristics of the sources of evidence used as eligibility criteria (e.g., years considered, language, and publication status), and provide a rationale. | 7 |
| Information sources* | 7 | Describe all information sources in the search (e.g., databases with dates of coverage and contact with authors to identify additional sources), as well as the date the most recent search was executed. | 6 |
| Search | 8 | Present the full electronic search strategy for at least 1 database, including any limits used, such that it could be repeated. | S2 |
| Selection of sources of evidence† | 9 | State the process for selecting sources of evidence (i.e., screening and eligibility) included in the scoping review. | 7 |
| Data charting process‡ | 10 | Describe the methods of charting data from the included sources of evidence (e.g., calibrated forms or forms that have been tested by the team before their use, and whether data charting was done independently or in duplicate) and any processes for obtaining and confirming data from investigators. | 9 |
| Data items | 11 | List and define all variables for which data were sought and any assumptions and simplifications made. | 8-9 |
| Critical appraisal of individual sources of evidence§ | 12 | If done, provide a rationale for conducting a critical appraisal of included sources of evidence; describe the methods used and how this information was used in any data synthesis (if appropriate). | na |
| Synthesis of results | 13 | Describe the methods of handling and summarizing the data that were charted. | na |
| **RESULTS** | | | |
| Selection of sources of evidence | 14 | Give numbers of sources of evidence screened, assessed for eligibility, and included in the review, with reasons for exclusions at each stage, ideally using a flow diagram. | na |
| Characteristics of sources of evidence | 15 | For each source of evidence, present characteristics for which data were charted and provide the citations. | na |
| Critical appraisal within sources of evidence | 16 | If done, present data on critical appraisal of included sources of evidence (see item 12). | na |
| Results of individual sources of evidence | 17 | For each included source of evidence, present the relevant data that were charted that relate to the review questions and objectives. | na |
| Synthesis of results | 18 | Summarize and/or present the charting results as they relate to the review questions and objectives. | na |
| **DISCUSSION** | | | |
| Summary of evidence | 19 | Summarize the main results (including an overview of concepts, themes, and types of evidence available), link to the review questions and objectives, and consider the relevance to key groups. | na |
| Limitations | 20 | Discuss the limitations of the scoping review process. | na |
| Conclusions | 21 | Provide a general interpretation of the results with respect to the review questions and objectives, as well as potential implications and/or next steps. | 9 |
| **FUNDING** | | | |
| Funding | 22 | Describe sources of funding for the included sources of evidence, as well as sources of funding for the scoping review. Describe the role of the funders of the scoping review. | 10 |

JBI = Joanna Briggs Institute; PRISMA-ScR = Preferred Reporting Items for Systematic reviews and Meta-Analyses extension for Scoping Reviews.

* Where *sources of evidence* (see second footnote) are compiled from, such as bibliographic databases, social media platforms, and Web sites.

† A more inclusive/heterogeneous term used to account for the different types of evidence or data sources (e.g., quantitative and/or qualitative research, expert opinion, and policy documents) that may be eligible in a scoping review as opposed to only studies. This is not to be confused with *information sources* (see first footnote).

‡ The frameworks by Arksey and O’Malley (6) and Levac and colleagues (7) and the JBI guidance (4, 5) refer to the process of data extraction in a scoping review as data charting*.*

§ The process of systematically examining research evidence to assess its validity, results, and relevance before using it to inform a decision. This term is used for items 12 and 19 instead of "risk of bias" (which is more applicable to systematic reviews of interventions) to include and acknowledge the various sources of evidence that may be used in a scoping review (e.g., quantitative and/or qualitative research, expert opinion, and policy document).

*From:* Tricco AC, Lillie E, Zarin W, O'Brien KK, Colquhoun H, Levac D, et al. PRISMA Extension for Scoping Reviews (PRISMAScR): Checklist and Explanation. Ann Intern Med. 2018;169:467–473. [doi: 10.7326/M18-0850](http://annals.org/aim/fullarticle/2700389/prisma-extension-scoping-reviews-prisma-scr-checklist-explanation).

**Search strategy**

**Ovid MEDLINE(R) ALL <1946 to October 02, 2023>**

<https://ovidsp.ovid.com/ovidweb.cgi?T=JS&NEWS=N&PAGE=main&SHAREDSEARCHID=1C9lhPoynJ4i47cp2apsZ5qNLGrGkt6y4A0Qw7YZSs4wEkoyaPbMkCXkvo0Zxpqyt>

1 Learning Health System/ 254

2 ("learning health*" adj3 (communit* or system* or network* or laboratory)).tw,kf. 1389

3 (learning adj1 collaborative).tw,kf. 1951

4 "rapid learn*".tw,kf. 541

5 Delivery of Health Care/ 118230

6 4 and 5 18

7 2 and 5 226

8 or/1-4,6-7 3852

9 Medical Informatics/ or Nursing Informatics/ or Informatics/ 15767

10 ((health or clinical) adj2 informatics*).tw,kf. 4841

11 data?driven.ti,ab. 16

12 (data* or informatic* or infomatic* or digital).tw,kf. 5417161

13 ("data driven" or "innovation hub").tw,kf. 19973

14 ("data collection" or "practice change*" or "continuous improvement*").tw,kf. 113100

15 Data Sets as Topic/ or Decisions Support Systems, Clinical/ 7398

16 or/9-15 5429926

17 8 and 16 1880

18 limit 17 to (english language and yr="2007 -Current") 1824

**Data extraction form**

1. First author, year
2. Source
   1. Article
   2. Report
   3. Website
   4. Informal discussion
   5. Other (specify)
3. Country
4. Institution name
5. Sectors involved (primary care, hospital, specialist care, long-term care, community/population/public health, industry, other)
6. Clinical population
7. Clinical population size if applicable
8. Reference to grounding framework or model
9. LHS keyword(s) (i.e., learning health system, learning collaboratory, community of practice, etc.)
10. Described goals/aims of LHS
11. Partners involved (select all that apply)
    1. Patients/caregivers
    2. Clinicians
    3. Health system leaders
    4. Health system operations
    5. Research
    6. Government/policymakers
    7. Other (specify)
12. LHS features described (select all that apply – do not need to describe)
    1. Core functionalities
    2. Analytic strategies
    3. Use of evidence
    4. Co-design and implementation approaches
    5. Evaluation and research integration
    6. Change management and governance structures
    7. Data and infrastructure sharing processes
    8. Knowledge sharing practices
    9. Training and capacity building approaches
    10. Health equity considerations
    11. Sustainability
    12. Other (specify)
13. Evaluation features
    1. Learning cycle vs. System level
    2. Approach
    3. Methods
    4. Question
    5. Theory of change/logic model
    6. Tools
    7. Outcomes
    8. Indicators and data sources
    9. Counterfactuals
    10. Theories or frameworks
    11. Personnel involved
    12. Governance
    13. Challenges identified
    14. Knowledge sharing or feedback
    15. System level metrics, indicators, or outcomes of growth, maturity, or success
14. Contextual factors relevant to design, implementation, operationalization of LHS (open text)
15. Mechanisms operating that contribute to design, implementation, operationalization of LHS (open text)

**Table S1. Extraction Criteria and Definitions**

| **Extraction Criterion** | **Definition** |
| --- | --- |
| Learning cycle vs. system level | Is the evaluation described occurring at the project level, the system level, or both? |
| Evaluation approach | “The overall framework for the evaluation and sets out what questions are selected and how the evaluation will be conducted.”^1^ (e.g., formative, summative, experimental, etc.). |
| Methods | “Provide what information should be collected, from which source(s) it should be collected, for what purpose it should be collected and how the collected data will be analyzed in order to answer the evaluation questions”^1^ (e.g., case study, randomized controlled trial, social network analysis). |
| Evaluation question | Note the evaluation question(s), if explicitly stated, and by whom the question was developed/posed. |
| Theory of change/Logic model | Note any hypotheses about the theory of change/programme theory or logic model that the authors state. |
| Tools | “The ways or channels for collecting the data required to answer the evaluation questions.”^1^ (e.g., EMR, surveys, qualitative in-depth interviews, document analysis). |
| Outcomes | Results of intervention, whether anticipated or unanticipated; positive, negative, or neutral. Can occur at the patient, provider, population, system level, etc.). |
| Indicators & data sources | Data (quantitative or qualitative) that indicate whether a change has occurred (e.g., clinical practice guideline targets reached, survey or performance scores, health services use, qualitative evidence of change). |
| Counterfactuals | Comparator scenario/group or ways in which “change from usual care/status quo” is evidenced, if any. Also describe how counterfactuals are established or defined. |
| Theories or frameworks | Theoretical or applied scaffolding which informs the logic or approach of evaluation. |
| Personnel involved | Internal or external personnel involved in designing, conducting, and interpreting/using results of evaluation, especially looking out for front-line staff and patient partners. |
| Evaluation governance | Governance or reporting structures involved in evaluation. |
| Challenges identified | Challenges identified (i.e., process challenges, data acquisition, resource challenges) |
| Knowledge-sharing or feedback | Description of how evaluation data are fed back into the system, “used” in LHS model, or shared externally. |
| Important contextual factors | Catch-all category for contextual elements not otherwise captured that may be important for assessment and interpretation of evaluation. |
| *System level only* Metrics, indicators, or outcomes of growth, maturity, or success | This may be more of a narrative, but we could look for discussion around how the LHS has grown, progressed, or advanced over time as it has incorporated more resources/personnel, added data collection mechanisms, conducted additional cycles... just a suggestion!! |

1. World Food Programme. *Technical Note: Evaluation Approaches, Methods and Data Collection Tools for Decentralized Evaluations.* Rome, Italy2021.

**Concepts drawn from:**

1. MRC Guidance: [A new framework for developing and evaluating complex interventions: update of Medical Research Council guidance | The BMJ](https://www.bmj.com/content/374/bmj.n2061)
2. Rainbow Framework (Better Evaluation): [Rainbow Framework - Rainbow Framework (betterevaluation.org)](https://www.betterevaluation.org/frameworks-guides/rainbow-framework)
